# Nontherapeutic INR After Hospital Discharge: A Repeated-Measures Analysis of Warfarin-Treated Patients and Potential Drug–Drug Interactions

**DOI:** 10.3390/clinpract16080136

**Published:** 2026-07-25

**Authors:** Kanthida Methaset, Pattamawan Kosuma, Arom Jedsadayanmata

**Affiliations:** 1 Division of Pharmacy Services, Thammasat University Hospital, Pathum Thani 12120, Thailand; kanthida@tu.ac.th; 2 Graduate Program in Drug Utilization and Health Outcomes Research, Faculty of Pharmacy, Thammasat University, Pathum Thani 12120, Thailand; 3 Faculty of Pharmacy, Thammasat University, Pathum Thani 12120, Thailand; pattamako@tu.ac.th

**Keywords:** hospital discharge, drug–drug interaction, international normalized ratio, prevalence, warfarin

## Abstract

**Background:** Warfarin remains widely used in specific clinical situations. Its management is complicated by multiple factors that affect anticoagulant response, particularly during the early period after hospital discharge. This study examined the prevalence, patterns, and factors associated with nontherapeutic international normalized ratio (INR) among patients discharged on warfarin from a tertiary-care hospital. **Methods:** Electronic health records of adult patients discharged home with warfarin who had at least one INR measurement within 90 days (*N* = 1222) were retrospectively analyzed. Nontherapeutic INR was defined as INR outside the therapeutic range: 2.5–3.5 for mitral valve replacement and 2.0–3.0 otherwise. All available INR measurements were included. Major warfarin potential drug–drug interactions (pDDIs) were defined as DDIs with major severity according to the Micromedex^®^ database. Factors associated with nontherapeutic INR were examined using repeated-measures generalized estimating equations (GEEs), with generalized linear mixed models (GLMMs) as confirmatory analyses. **Results:** Of 3704 INR measurements within 90 days after discharge, 49.4% were subtherapeutic, while 30.5% were therapeutic and 20.1% were supratherapeutic. The proportion of therapeutic INR values did not show a substantial improvement over time. In GEEs, discharge from surgical service (adjusted odds ratio (aOR) 1.24, 95%CI: 1.05–1.48, *p* = 0.014) and presence of major warfarin pDDIs at discharge (aOR 1.36, 95%CI: 1.11–1.67, *p* = 0.003) were associated with nontherapeutic INR. GLMM analyses produced consistent results with the GEE model. **Conclusions:** Suboptimal INR control was prevalent within 90 days post-discharge. Discharge from surgical services and presence of major warfarin pDDIs at discharge were associated with nontherapeutic INRs. Major warfarin pDDIs may serve as markers of medication complexity at discharge and may help identify patients requiring closer anticoagulation monitoring.

## 1. Introduction

Warfarin continues to play a crucial role in preventing and treating thromboembolic conditions, despite the increasing use of direct oral anticoagulants (DOACs) in clinical practice. Current guidelines recommend warfarin for patients with mechanical prosthetic heart valves and in situations where DOAC therapy is unsuitable or contraindicated [1,2]. Warfarin offers the advantage of individualized dose titration based on international normalized ratio (INR) monitoring, allowing clinicians to adjust its anticoagulant effect. Because DOACs undergo partial renal elimination, their use may be limited in patients with advanced chronic kidney disease. Moreover, warfarin remains a cost-effective option, particularly in resource-limited healthcare systems where the higher expense of DOACs may pose a barrier to access [3,4,5].

Warfarin therapy is challenging to manage because of its narrow therapeutic index and marked interpatient variability in response. Several factors can contribute to fluctuations in anticoagulation control, including patient genetics and comorbidities, variability in adherence, dietary influences, and potential drug–drug interactions (pDDIs) [6,7,8,9,10,11,12]. Such variability often results in nontherapeutic INR levels, complicating long-term management. The transition from hospital to home is a particularly vulnerable period, as patients are frequently discharged with multiple medications to manage coexisting conditions, which may contribute to nontherapeutic INR [13]. In a study of patients with atrial fibrillation, nearly half of the INR measurements were outside the target range [14,15]. Additionally, environmental factors, such as modifications to the patient’s diet, changes in daily activity, and the initiation or discontinuation of interacting therapies, may contribute to INR fluctuations during early follow-up [7,8,9,10,11].

Maintaining INR within the therapeutic range is critical, as deviations are strongly associated with adverse outcomes such as bleeding or thromboembolic complications [14,15,16,17,18,19,20]. Understanding the prevalence and patterns of nontherapeutic INR values and associated factors is essential for improving the quality of care for patients discharged on warfarin therapy [9,21,22]. However, few studies have specifically evaluated INR control during the early transition from hospital discharge to outpatient follow-up using repeated INR measurements. In our previous study of patients discharged on warfarin, major warfarin pDDIs were highly prevalent at discharge, affecting more than 80% of patients, with polypharmacy identified as a key determinant of pDDI burden [23]. However, that study focused on discharge prescribing and did not evaluate subsequent INR control. The present study therefore extends this work by exploring discharge-related factors, including major warfarin pDDIs, and their association with repeated INR measurements during the 90-day post-discharge period.

The present study aimed to describe the prevalence and patterns of INR control within 90 days after hospital discharge among warfarin-treated patients and to determine factors associated with nontherapeutic INR using repeated INR measurements at outpatient follow-up. We hypothesized that clinical- and medication-related factors at discharge would be associated with nontherapeutic INR during early follow-up.

## 2. Methods

### 2.1. Study Setting and Participants

This retrospective cohort study was part of a larger study conducted at an 800-bed tertiary-care teaching hospital in central Thailand. The larger population (*N* = 1667) was studied to determine the prevalence of warfarin pDDIs at hospital discharge [23]. Our previous publication focused on the prevalence and determinants of warfarin major pDDIs identified at hospital discharge [23]. In contrast, the present study addresses a separate research question by restricting the analysis to patients with available post-discharge INR measurements and evaluating repeated INR control during the 90-day transition from hospital discharge to outpatient follow-up. The study hospital and its clinics serve as a medical training center for health professional students, residents, and fellows, and provide a wide range of specialized services, including cardiology, neurology, oncology, and surgery. It serves as a referral center for regional hospitals, providing advanced procedures and specialized care.

Participants were identified from electronic health records (EHRs) of patients who were admitted for at least 24 h and discharged home on warfarin. The study period covered 1 January 2019 to 31 December 2022. Eligible patients were aged 18 years or older at the time of discharge and were prescribed warfarin together with at least one other medication. The analysis was limited to patients discharged from medical or surgical services, including general medicine, cardiology, pulmonology, and internal medicine subspecialties, as well as general and specialized surgical departments such as orthopedics and cardiovascular surgery. For individuals with more than one hospitalization during the study period, only the first admission was considered as the index hospitalization to ensure each patient contributed a single, unique baseline profile at discharge and to avoid the artificial duplication of baseline covariates in the regression models. To evaluate factors associated with INR control, study participants had to meet the previously mentioned criteria and have at least 1 INR measurement within 90 days of discharge. This requirement ensured that data were available to determine the prevalence, pattern, and factors associated with INR control over time. Because this was a retrospective study using routinely collected data, no formal a priori sample size calculation was performed. All eligible patients who met the inclusion criteria during the study period were included. The final analytic sample included 1222 patients contributing 3704 INR measurements, and seven pre-specified independent variables were included in the multivariable models.

### 2.2. Variables and Data Collection

Data were collected from EHRs, pharmacy, and laboratory databases, and linked using a unique, de-identified code for each patient. The Information Technology Department of the study hospital provided the final dataset for research purposes only. The authors did not have access to information that could identify individual participants during or after data collection. Key variables included patient demographics, discharge services, admission and discharge dates, principal diagnosis, comorbidities, medication orders, and INR results.

Warfarin pDDIs were identified using the Micromedex^®^ Drug Interaction database, without restriction by documentation level. Micromedex^®^ classifies warfarin pDDIs into major, moderate, or minor severity. For this study, we further grouped them into two categories: major and non-major. Major pDDIs, as defined by Micromedex^®^, are interactions that may be life-threatening or require medical intervention to prevent or mitigate serious adverse effects. In the analysis, patients with at least one major pDDI were classified as having a major warfarin pDDI (coded as 1), whereas those with only moderate, minor, or no pDDIs were classified as having no major pDDI (coded as 0). This dichotomization was applied to highlight interactions of greatest clinical relevance and ensure statistical model stability during multivariable regression.

Polypharmacy was defined as the concurrent use of five or more medications at the time of hospital discharge. This threshold was selected to maintain methodological consistency with our previous work in this clinical population and aligns with widely accepted epidemiological and World Health Organization standards for evaluating medication burden [24,25].

INR control was assessed according to indication-specific therapeutic ranges: INR 2.5–3.5 for patients with mitral valve replacement (MVR) and 2.0–3.0 for other indications. Each INR measurement was coded as nontherapeutic (1) if it fell outside the indication-specific therapeutic range, and therapeutic (0) if it fell within the range. Nontherapeutic INRs were further classified descriptively as subtherapeutic and supratherapeutic. Subtherapeutic INRs were defined as <2.5 (for MVR) or <2.0 (for other indications), and supratherapeutic INRs as >3.5 (MVR) or >3.0 (others). Because many patients had multiple concomitant pDDIs with potentially opposing or uncertain effects on INR, INR control was analyzed as a binary outcome (therapeutic versus nontherapeutic) to reflect overall failure to remain within the therapeutic range, rather than as separate subtherapeutic and supratherapeutic outcomes.

INR control was evaluated at the individual measurement level rather than using longitudinal metrics such as Time in Therapeutic Range (TTR). This approach was selected because TTR calculations can be mathematically unstable during the short-term (90-day) post-discharge transition period, where testing intervals are highly irregular and dose titrations are frequent. Furthermore, evaluating discrete measurements allows the model to capture acute episodes of nontherapeutic readings that require immediate clinical intervention during this high-risk care transition. For rare instances where multiple INR measurements occurred on the same day, the second result was selected to capture the final, clinically verified confirmatory value.

### 2.3. Statistical Analyses

To examine the prevalence and patterns of INR control, each INR record was classified as subtherapeutic, therapeutic, or supratherapeutic, and summarized in 10-day intervals over the 90-day follow-up period. This 10-day interval was selected to align with the typical clinical frequency of early post-discharge INR monitoring (generally every 7 to 14 days), providing a practical framework for presenting the descriptive patterns of INR control over time. Results were presented as the number and percentage of INR measurements within each interval. In addition, the first seven INR measurements after discharge were summarized to visualize patterns of control by testing order. A kernel density plot was used as an exploratory descriptive analysis to visualize the distribution of observed INR measurements. The plot was not used for inferential analysis and is presented in the Supplementary Materials.

Generalized estimating equations (GEEs) with a binomial family and logit link were used as the primary repeated-measures model to estimate population-averaged associations between covariates and nontherapeutic INR while accounting for repeated INR measurements within patients. Generalized linear mixed models (GLMMs) with patient-specific random intercepts were fitted as confirmatory analyses. Both models included the same prespecified covariates. Additional technical details on working correlation selection, handling of repeated measurements, use of robust standard errors, handling of missing data, and multicollinearity assessment are provided in the Supplementary Materials under the Supplementary Methods Section.

As part of the sensitivity analyses, we conducted three additional evaluations. First, we re-estimated the associations between covariates and nontherapeutic INR using GEEs and GLMMs, restricting INR measurements to the 30- and 60-day windows following discharge, rather than the 90-day window used in the primary analysis. Second, we repeated the analyses by limiting the INR testing sequence to the first three and seven INR measurements, rather than including all available INR results. Third, we broadened the definition of the therapeutic INR range in two steps to assess the consistency of the findings under more permissive definitions: first, to 2.30–3.70 for patients with MVR and 1.80–3.20 for those without MVR, and further to 2.00–4.00 for patients with MVR and 1.50–3.50 for those without MVR. The minor expansion (±0.2) captures near-therapeutic variations where active dose adjustments are routinely withheld in stable clinical practice. The broader expansion (±0.5) aligns with safety and clinical action thresholds, outside of which corrective interventions are more likely to be considered.

All statistical analyses were performed using Stata Statistical Software: Release 18 (StataCorp, College Station, TX, USA). All hypothesis tests were two-sided, with a statistical significance level of 0.05.

### 2.4. Ethical Considerations

The Human Research Ethics Committee of Thammasat University approved the study on 6 April 2023 (approval number: COA 022/2566, research project code: 66PH033). Informed consent was unnecessary due to the retrospective nature of the data collection, which posed minimal risk to participants. All data were analyzed anonymously. This study was conducted in accordance with the Declaration of Helsinki. Data access and handling followed institutional and ethical guidelines to ensure patient confidentiality.

## 3. Results

### 3.1. Baseline Characteristics of Study Participants

Table 1 presents the baseline characteristics of the 1222 patients at discharge, stratified by the presence of major warfarin pDDIs. Among the cohort, 994 (81.3%) were discharged with at least one major pDDI. Overall, the average number of major warfarin pDDIs per patient at discharge was 1.53 ± 1.15. Male sex and polypharmacy were more prevalent in patients who were discharged with major pDDIs. The prevalence of atrial fibrillation and mitral valve stenosis was higher in those discharged without major pDDIs. The list of warfarin-interacting medications with major severity and proportions is shown in Supplementary Table S1.

Table 1 also provides details of the INR measurements after discharge. The median time to the first INR measurement was one day shorter (8 days versus 9 days), while the median INR values were slightly lower (2.0 versus 2.2) among patients with major warfarin pDDIs.

### 3.2. Distribution of Post-Discharge INR Control

**Figure 1:**
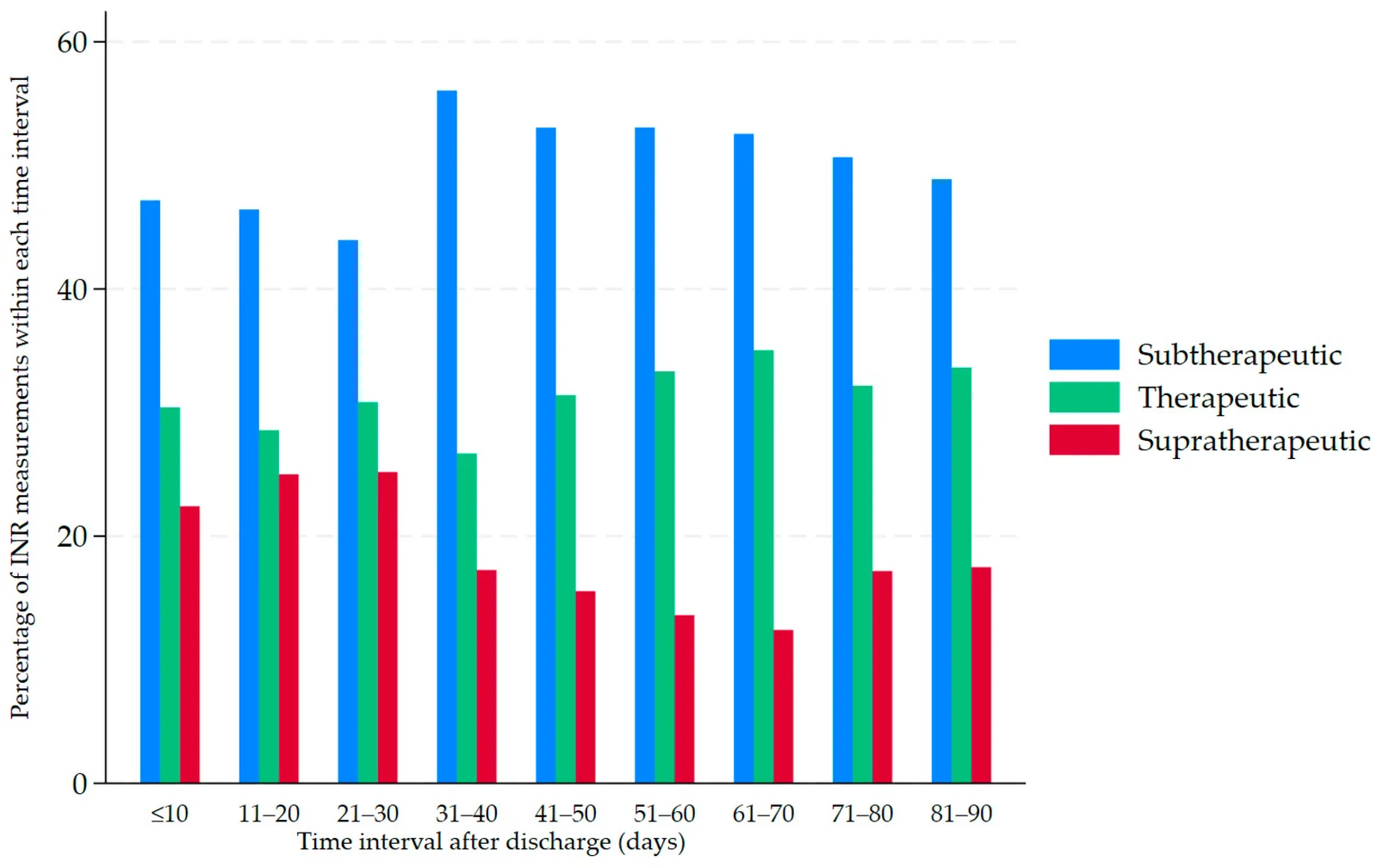
Distribution of INR categories during the 90 days after hospital discharge. Therapeutic ranges were defined as 2.5–3.5 for mitral valve replacement and 2.0–3.0 for all other conditions. Bars represent the proportion of INR measurements that were subtherapeutic, therapeutic, or supratherapeutic within each 10-day interval. Figure 1 provides visual complements to Table 2.

Table 2 summarizes INR measurements and categories—subtherapeutic, therapeutic, and supratherapeutic—according to indication-specific therapeutic ranges. For 3704 INR measurements, 901 (24.3%) occurred within the first 10 days after discharge, while the proportion decreased to <10% in each 10-day interval beyond 30 days. Overall, 49.4% of INR values were subtherapeutic, 30.5% were therapeutic, and 20.1% were supratherapeutic. These proportions remained relatively stable across time intervals, with subtherapeutic values consistently comprising the largest proportion (44–56%), therapeutic values fluctuating between 27% and 35%, and supratherapeutic values between 13% and 25% (Figure 1).

**Figure 2:**
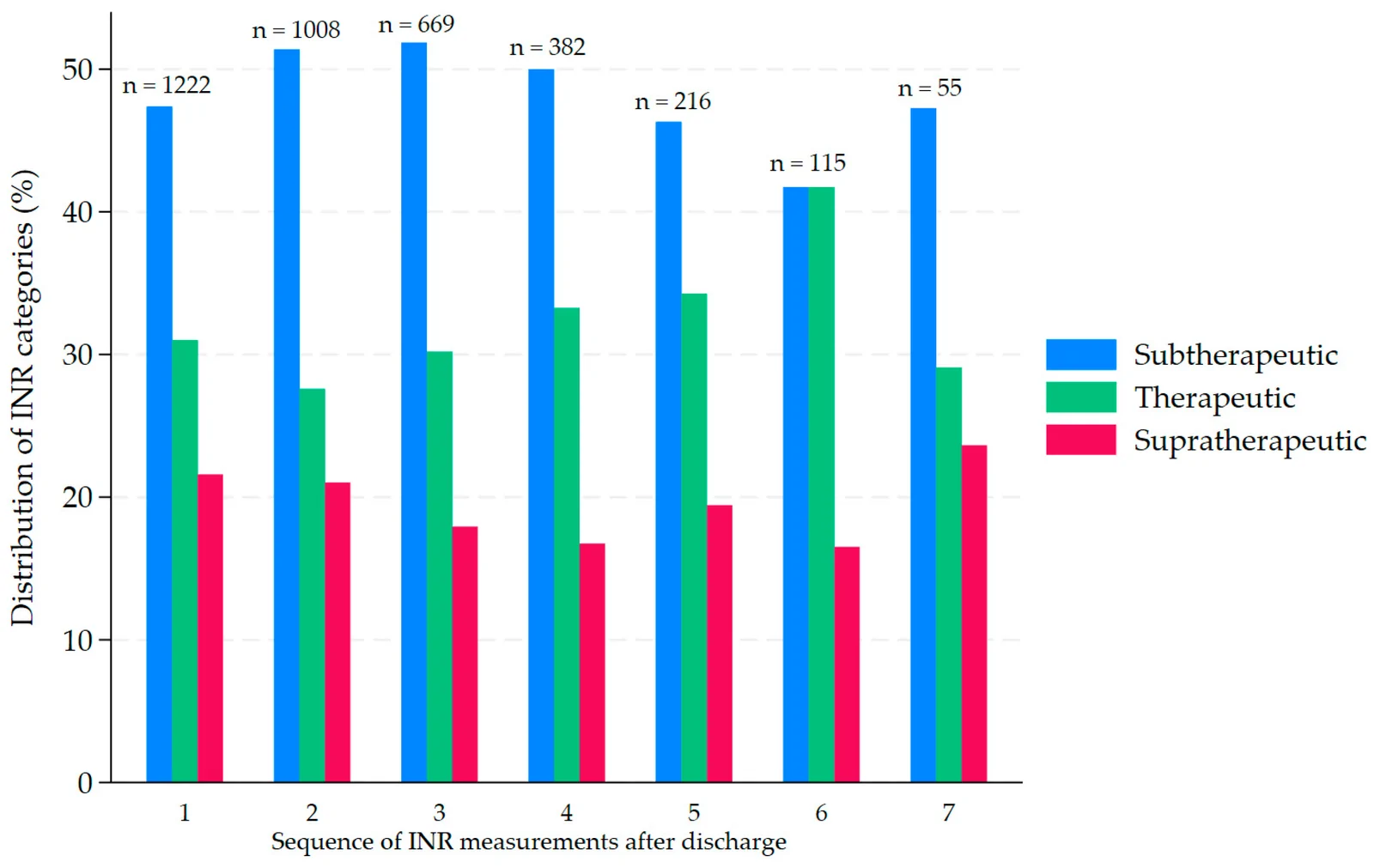
Distribution of INR categories by consecutive order of INR testing. The *n* above each group of bars represents the total number of INR measurements in each INR testing sequence. The first seven INR measurements within 90 days after discharge are presented.

A similar pattern was observed when results were examined by the order of INR testing: subtherapeutic values predominated across the first seven INR measurements (ranging from 47% to 52%), while therapeutic values accounted for 27–42% and supratherapeutic values for 16–25% (Figure 2). Although the number of patients contributing to subsequent INR tests declined, the category distribution remained consistent across successive measurements. When examining the first INR measurement for each patient, 50% were subtherapeutic, 30% were therapeutic, and 20% were supratherapeutic, consistent with the distribution observed across all 90-day INR measurements.

The distribution of INR values stratified by patient characteristics is shown in Supplementary Figure S1. Across most subgroups, the density curves were largely overlapping, with most INR values clustering in the subtherapeutic range. Small shifts were observed for patients discharged from surgical services and those with major warfarin pDDIs at discharge, but the overall distributions remained broadly overlapping. To further distinguish the direction of INR deviation, the distributions of subtherapeutic, therapeutic, and supratherapeutic INR values across baseline strata are presented in Supplementary Table S2.

### 3.3. Factors Associated with INR Control After Discharge

GEEs and GLMMs were used to identify factors associated with INR control, defined as either within the therapeutic range (therapeutic) or outside it (nontherapeutic). In GEEs (Table 3), being discharged from a surgical service was associated with nontherapeutic INR after discharge (adjusted odds ratio (aOR) 1.24, 95%CI: 1.05–1.48, *p* = 0.014). The presence of major warfarin pDDIs at discharge was associated with nontherapeutic INR in univariable analysis and remained significant after adjustment for other covariates (aOR 1.36, 95% CI: 1.11–1.67, *p* = 0.003). Similar associations were observed in the GLMM model with each patient as a random intercept (Table 4). Other factors, such as age, polypharmacy, LOS, and the number of comorbidities, were not significantly associated with nontherapeutic INR in either model.

To evaluate the consistency of the findings, sensitivity analyses were performed using both GEEs and GLMMs. Sensitivity analyses showed directionally consistent associations between major warfarin pDDIs at discharge and nontherapeutic INR across alternative follow-up windows, restricted numbers of INR measurements, and expanded therapeutic INR definitions, although estimates were attenuated under the broadest INR definition. Detailed model results and summary of sensitivity analyses are presented in Supplementary Tables S3–S9.

## 4. Discussion

The present study aimed to determine the prevalence, patterns, and factors associated with INR control within 90 days after discharge among warfarin-treated patients. Our results showed that suboptimal INR control was prevalent after discharge, with nearly 70% of INR measurements falling outside the therapeutic range (subtherapeutic or supratherapeutic). Beyond describing this burden, our repeated-measures analyses showed that discharge from surgical services and the presence of major warfarin pDDIs at discharge were associated with nontherapeutic INR during follow-up. These findings suggest that both factors may help identify patients who require closer INR monitoring after discharge.

We observed that about 30% of INR values during the first 90 days after discharge were within the therapeutic range, a pattern also observed for the first INR measurement after discharge (Figure 2). This finding is broadly consistent with prior post-discharge studies: Jackson et al. reported that by day 8 after discharge, 33% of INRs were subtherapeutic and 26% were supratherapeutic, while another study found that the first post-discharge INR was 23% subtherapeutic and 26% supratherapeutic [26,27]. Although not specific to the immediate post-discharge period, van Walraven et al. similarly found that prior hospitalization was associated with increased risk of both subtherapeutic and supratherapeutic INR values during follow-up [28,29]. Taken together, these findings suggest that suboptimal INR control is common during the early post-discharge period.

Approximately 50% of the INR records in our study were subtherapeutic, including those from early and subsequent measurements (Figure 2). This predominance of subtherapeutic INR values is likely multifactorial [9,21,22,30]. Prior studies have reported that Asian patients often require lower warfarin doses to achieve INR levels comparable with Western populations [31,32,33]. In addition, cautious anticoagulation practices may occur in clinical settings where bleeding risk is a major concern. Previous studies in Thai patients have suggested that lower-intensity anticoagulation may be considered in certain clinical contexts. One study in patients with mechanical valve replacement reported an optimal INR range of 2.0–3.4 [34], where as another study in patients with atrial fibrillation suggested that a lower warfarin dose may be optimal [35]. However, provider-intended INR targets and prescribing rationale were not directly assessed in the present study. Whether the predominance of subtherapeutic INR values reflects biological dose requirements, intentional anticoagulation practices, or other care-process factors warrants examination in future studies.

Multivariable analyses identified major warfarin pDDIs as an important factor associated with nontherapeutic INR in the present study. Patients with such interactions were approximately 36–37% more likely to have an INR outside the therapeutic range compared with those who were not. This association was similarly observed in both the population-averaged GEE model and the subject-specific GLMM model. Because these models have different interpretations, the GLMM analysis was treated as confirmatory rather than equivalent to the GEE analysis. Given the observational design and discharge-level assessment, however, this result should be interpreted as indicating a higher-risk clinical profile rather than a drug-specific causal mechanism. Major pDDIs were analyzed as a single binary variable because the objective was to evaluate the overall burden of potentially high-risk prescribing at discharge, rather than to isolate individual causal pathways. This approach was considered appropriate because many patients had multiple concurrent major pDDIs at discharge [23], and individual interactions may have effects on INR that are opposing, uncertain, or not mediated through INR.

The association between major warfarin pDDIs and nontherapeutic INR should be interpreted in the context of the heterogeneity of the composite pDDI. Major pDDIs do not represent a single biological mechanism; some may affect INR, whereas others may increase bleeding risk without directly altering INR. For example, omeprazole accounted for 40% of major warfarin pDDIs in the present study, although its effect on INR remains debated [36]. Other common major pDDIs, such as aspirin, enoxaparin, and clopidogrel, may increase bleeding risk without directly affecting INR. Therefore, the major pDDI variable is best interpreted as a discharge-level marker of medication-related complexity and anticoagulation-related risk rather than as a biologically homogeneous exposure with a uniform directional effect on INR [11,17,36,37].

The high prevalence of major pDDIs should be interpreted in the context of database-based interaction screening, which identifies potential rather than clinically confirmed interactions [23]. Although some overclassification is possible because patient-specific clinical relevance was not adjudicated, the observed prevalence is plausible in a tertiary-care discharge cohort with substantial polypharmacy and comorbidities. Thus, the finding may reflect both the sensitivity of database-based screening and the complexity of medications in transitional-care pharmacotherapy.

Surgical discharge was associated with a higher likelihood of nontherapeutic INR, possibly reflecting the complexity of postoperative anticoagulation management, perioperative medication changes, and differences in discharge or follow-up processes. Further studies incorporating perioperative clinical details, medication changes, and care-process measures are needed to clarify this association.

Although polypharmacy was a key determinant of major pDDI burden in our previous study [23], medication count alone was not independently associated with nontherapeutic INR in the present analysis. This difference is plausible because pDDI burden is directly related to the number of concomitant medications, whereas INR control depends on multiple factors [21,22,30,37,38], including the specific interacting drugs, dose adjustment, adherence, diet, and post-discharge medication changes. Medication count may therefore be useful for identifying patients at risk of pDDIs, but less informative for identifying post-discharge INR instability. In contrast, major warfarin pDDIs may serve as markers of medication complexity at discharge and help identify patients who may require closer post-discharge INR monitoring.

The sensitivity analysis showed that when INR measurements were restricted to within 30 days after discharge, the associations between discharge from surgery, major pDDIs, and nontherapeutic INR values were no longer statistically significant (Supplementary Tables S3 and S4). This attenuation may be explained by reduced statistical power. Because both GEEs and GLMMs account for repeated measurements, the number of available INR observations affects model precision. Limiting the analysis to a shorter follow-up period substantially decreased the number of measurements per participant. This may have resulted in wider confidence intervals and loss of statistical significance despite directionally consistent estimates. However, potential clinical influences on INR control during the early post-discharge period cannot be entirely excluded.

From a practice perspective, the presence of major warfarin pDDIs at discharge and discharge from surgical services may help identify patients who require more structured post-discharge anticoagulation support. Although preemptive dose adjustment has been suggested for selected warfarin pDDIs, the clinical impact of such strategies remains uncertain [39]. Therefore, discharge processes for these patients could emphasize pharmacist-led medication reconciliation, explicit documentation of interacting medications and intended INR targets, scheduling of early INR follow-up before discharge, and direct communication of anticoagulation plans to outpatient providers or referral hospitals [40,41]. These strategies should be interpreted as risk-management implications from the present exploratory analysis, rather than as interventions proven to improve clinical outcomes.

This study has several limitations. First, its retrospective observational design limits causal inference, and unmeasured factors such as health literacy, dietary vitamin K intake, alcohol use, medication adherence, socioeconomic status, and access to anticoagulation clinics could not be captured. Second, warfarin pDDIs were assessed only at discharge and treated as fixed baseline covariates during the 90-day follow-up period, so post-discharge medication changes, dose adjustments, and the duration of concomitant therapy were not accounted for. This may have introduced misclassification of pDDI status during follow-up and attenuated the observed associations. Third, the requirement for at least one outpatient INR measurement may have introduced selection bias by excluding patients monitored outside the study hospital or not monitored after discharge. Fourth, the binary INR outcome did not capture the magnitude or direction of INR deviation, and the strict therapeutic ranges may have overestimated the prevalence of suboptimal control. Finally, clinical outcomes such as bleeding and thromboembolic events were not analyzed, and the single-center setting may limit generalizability.

## 5. Conclusions

In this single-center retrospective study, nontherapeutic INR values were common during the first 90 days after hospital discharge among patients receiving warfarin. Discharge from surgical services and the presence of major warfarin pDDIs at discharge were associated with nontherapeutic INR during follow-up. These findings should be interpreted as exploratory and hypothesis-generating, as major pDDIs represent discharge-level markers of medication-related complexity rather than biologically homogeneous exposures with a uniform directional effect on INR. Patients discharged with major warfarin pDDIs may represent a clinically complex subgroup requiring closer post-discharge anticoagulation monitoring. Prospective multicenter studies incorporating time-varying medication and pDDI status, TTR, and clinical outcomes such as bleeding and thromboembolic events are needed to clarify the clinical impact of discharge-related medication complexity on warfarin management after hospitalization.

## Figures and Tables

**Table 1:** Baseline characteristics of patients at discharge (*N* = 1222).

Characteristics	Presence of Major Warfarin pDDIs		
	No *n* (%)	Yes *n* (%)	*p*-Values *
Number of patients (%)	228 (18.7)	994 (81.3)	-
Age in years (mean ± SD)	62.19 ± 16.52	63.76 ± 15.27	0.167
Age ≥ 65 years	109 (47.8)	518 (52.1)	0.241
Male	85 (37.3)	485 (48.8)	0.002
**Discharge service**			<0.001
Medicine	151 (66.2)	516 (51.9)	
Surgery	77 (33.8)	478 (48.1)	
LOS in days (mean ± SD)	11.70 ± 13.82	13.09 ± 14.27	0.182
LOS ≥ 10 days	99 (43.4)	475 (47.8)	0.234
Polypharmacy (≥5 medications at discharge)	178 (78.1)	938 (94.4)	<0.001
Number of comorbidities (mean ± SD)	4.75 ± 2.71	4.61 ± 2.66	0.457
Number of comorbidities ≥ 5	109 (47.8)	463 (46.6)	0.738
**Comorbidities**			
Atrial fibrillation	109 (47.8)	370 (37.2)	0.003
Chronic heart failure	37 (16.2)	129 (13.0)	0.196
Chronic kidney disease	28 (12.3)	121 (12.2)	0.964
Coronary artery disease	15 (6.6)	208 (20.9)	<0.001
Dyslipidemia	51 (22.4)	253 (25.5)	0.331
Diabetes mellitus	45 (19.7)	246 (24.7)	0.109
Hypertension	92 (40.4)	480 (48.3)	0.030
Ischemic stroke	23 (10.1)	110 (11.1)	0.669
Mitral valve stenosis	31 (13.6)	57 (5.7)	<0.001
Replacement of mechanical mitral valve	11 (4.8)	44 (4.4)	0.794
Replacement of mechanical aortic valve	6 (2.6)	52 (5.2)	0.096
Venous thromboembolism	3 (1.3)	19 (1.9)	0.542
INR measurements			
No. of INR measurements (mean ± SD)	2.82 ± 1.53	3.11 ± 1.79	0.024
No. of INR measurements (median (IQR))	3 (2–4)	3 (2–4)	0.075
Days to first INR measurement (mean ± SD)	14.67 ± 16.71	10.35 ± 9.98	<0.001
Days to first INR measurement (median (IQR))	9 (5–15)	8 (5–12)	<0.001
INR values over a 90-day period (median (IQR))	2.2 (1.6–2.9)	2.0 (1.5–2.8)	<0.001

* Independent *t*-test for quantitative variables, Chi-square test for categorical variables, and Wilcoxon rank-sum test for the variables presented as median (IQR). pDDIs, potential drug–drug interactions; IQR, interquartile range; LOS, length of stay.

**Table 2:** Distribution of INR category after discharge (*N* = 1222).

INR Category	Time Interval After Discharge (Days)									Total
	1–10	11–20	21–30	31–40	41–50	51–60	61–70	71–80	81–90	
Subtherapeutic *n* (%)	425 (47.2)	312 (46.4)	178 (44.0)	250 (56.0)	174 (53.1)	121 (53.1)	144 (52.6)	115 (50.6)	109 (48.9)	1828 (49.4)
Therapeutic *n* (%)	274 (30.4)	192 (28.5)	125 (30.9)	119 (26.7)	103 (31.4)	76 (33.3)	96 (35.0)	73 (32.2)	75 (33.6)	1133 (30.5)
Supratherapeutic *n* (%)	202 (22.4)	168 (25.1)	102 (25.1)	77 (17.3)	51 (15.5)	31 (13.6)	34 (12.4)	39 (17.2)	39 (17.5)	743 (20.1)
Total INR measurements *n* (%)	901 (24.3)	672 (18.1)	405 (11.0)	446 (12.0)	328 (8.9)	228 (6.2)	274 (7.4)	227 (6.1)	223 (6.0)	3704 (100)

Subtherapeutic, INR below 2.0 (or below 2.5 for MVR); therapeutic, INR 2.0–3.0 (2.5–3.5 for MVR); supratherapeutic, INR above 3.0 (or above 3.5 for MVR). Percentages represent the proportion of total INR measurements within each time interval.

**Table 3:** Factors associated with nontherapeutic INRs measured within 90 days post-discharge in the GEE analyses * (*N* = 1222).

Covariates	Univariable Analysis		Multivariable Analysis	
	Crude OR (95% CI)	*p*-Values	Adjusted OR (95%CI)	*p*-Values
**Sex**				
Female	1.00		1.00	
Male	1.06 (0.90–1.24)	0.493	1.01 (0.86–1.19)	0.912
**Age**				
<65 years	1.00		1.00	
≥65 years	1.09 (0.93–1.27)	0.316	1.08 (0.92–1.28)	0.346
**Discharge service**				
Medicine	1.00		1.00	
Surgery	1.26 (1.07–1.48)	0.005	1.24 (1.05–1.48)	0.014
**No. of comorbidities**				
<5	1.00		1.00	
≥5	0.94 (0.80–1.10)	0.423	0.96 (0.81–1.14)	0.654
**Polypharmacy**				
No	1.00		1.00	
Yes	1.42 (1.05–1.90)	0.022	1.33 (0.98–1.82)	0.070
**Length of hospital stay**				
<10 days	1.00		1.00	
≥10 days	1.08 (0.92–1.26)	0.374	1.04 (0.88–1.22)	0.676
**Major warfarin pDDIs**				
No	1.00		1.00	
Yes	1.43 (1.20–1.77)	<0.001	1.36 (1.11–1.67)	0.003

* The GEE model used a binomial distribution with a logit link, with standard errors adjusted for patient clustering. An independent working correlation structure was selected based on the lowest QIC value.

**Table 4:** Factors associated with nontherapeutic INRs measured within 90 days post-discharge in the GLMM analyses * (*N* = 1222).

Covariates	Univariable Analysis		Multivariable Analysis	
	Crude OR (95% CI)	*p*-Values	Adjusted OR (95% CI)	*p*-Values
**Sex**				
Female	1.00		1.00	
Male	1.06 (0.88–1.27)	0.537	0.99 (0.83–1.19)	0.957
**Age**				
<65 years	1.00		1.00	
≥65 years	1.08 (0.90–1.29)	0.408	1.19 (0.98–1.45)	0.073
**Discharge service**				
Medicine	1.00		1.00	
Surgery	1.31 (1.10–1.58)	0.003	1.28 (1.05–1.55)	0.012
**No. of comorbidities**				
<5	1.00		1.00	
≥5	0.92 (0.77–1.10)	0.337	0.93 (0.77–1.12)	0.441
**Polypharmacy**				
No	1.00		1.00	
Yes	1.47 (1.05–2.04)	0.024	1.31 (0.92–1.87)	0.130
**Length of hospital stay**				
<10 days	1.00		1.00	
≥10 days	1.13 (0.94–1.35)	0.187	1.09 (0.91–1.31)	0.357
**Major warfarin pDDIs**				
No	1.00		1.00	
Yes	1.49 (1.19–1.86)	<0.001	1.37 (1.09–1.73)	0.008

* The GLMM was specified with a binomial distribution and a logit link function. Random intercepts for individual patients were included to account for the within-patient correlation of INR measurements.

## Data Availability

The data underlying this article were provided by the Thammasat University Hospital with permission. Data will be shared with the corresponding author upon request, provided permission is obtained from Thammasat University Hospital.

## Supplementary Materials

The following supporting information can be downloaded at: https://www.mdpi.com/article/10.3390/clinpract16080136/s1. Table S1. Prevalence of major warfarin pDDIs (*N* = 1222). Table S2. Distribution of post-discharge INR categories from repeated INR measurements stratified by baseline characteristics (*N* = 3704 total INR measurements). Table S3. Sensitivity analyses of the association between covariates and INR control using the GEE model within 30, 60, and 90 days post-discharge. Table S4. Sensitivity analyses of the association between covariates and INR control using the GLMM model within 30, 60, and 90 days post-discharge. Table S5. Sensitivity analyses of the association between covariates and INR control using the GEE model based on the number of INR measurements (first 3, first 7, and all INR measurements). Table S6. Sensitivity analyses of the association between covariates and INR control using the GLMM model based on the number of INR measurements (first 3, first 7, and all INR measurements). Table S7. Sensitivity analyses of the association between covariates and INR control using the GEE model under expanded therapeutic INR definitions. Table S8. Sensitivity analyses of the association between covariates and INR control using the GLMM under expanded therapeutic INR definitions. Table S9. Summary sensitivity analyses for the association between major warfarin pDDIs at discharge and nontherapeutic INR. Figure S1: Probability density plots of INR values stratified by baseline characteristics. Panel A shows sex (female vs male); Panel B, age group; Panel C, discharge service; Panel D, number of comorbidities; Panel E, length of hospital stay; Panel F, presence of polypharmacy; and Panel G, presence of major warfarin pDDIs. Vertical dashed lines indicate the INR values of 2.0–3.0.

## Abbreviations

The following abbreviations are used in this manuscript:

GEE	Generalized estimating equation
GLMM	Generalized linear mixed model
INR	International normalized ratio
LOS	Length of hospital stay
MVR	Mitral valve replacement
OR	Odds ratio
pDDIs	Potential drug–drug interactions
QIC	Quasi-likelihood under the independence model criterion
