# Supplementary material for: Nontherapeutic INR After Hospital Discharge: A Repeated-Measures Analysis of Warfarin-Treated Patients and Potential Drug–Drug Interactions
Source: Clin Pract. 2026 Jul 25;16(8):136. doi: 10.3390/clinpract16080136 (PMC13510403; doi:10.3390/clinpract16080136)
Supplement: Supplementary file 1 [file clinpract-16-00136-s001.zip › clinpract-4314379-supplementary.pdf]

SUPPLEMENTARY MATERIALS

Supplementary methods: Statistical analysis

Generalized Estimating Equation (GEE)

GEE was employed to identify factors associated with nontherapeutic INR after discharge. We first conducted univariable analysis to explore the association between each variable and the nontherapeutic INR. All variables were then entered into multivariable analyses to determine their independent association with the nontherapeutic INR, defined as whether the INR remained within the therapeutic range at each follow-up visit. Independent variables included patient age, sex, number of documented comorbidities, discharge service, polypharmacy (≥5 concurrent medications), length of stay (LOS) during the index hospitalization, and the presence of warfarin major pDDIs. The independent variables included in the multivariable models were pre-specified based on clinical relevance. Univariable statistical significance was not used as the sole criterion for variable inclusion because the purpose of the analysis was to estimate adjusted associations between selected clinical variables and nontherapeutic INR.

GEE models with a binomial family and logit link were used to estimate population-averaged associations between covariates and nontherapeutic INR while accounting for repeated INR measurements within patients. Candidate working correlation structures were compared using QIC, and the independent structure yielded the lowest QIC. The independent structure was selected for the primary GEE model and treated as a working assumption for estimation rather than as evidence that repeated INR measurements within patients were biologically independent. Because the working correlation structure in GEE is an assumption, robust Huber–White sandwich standard errors, clustered at the patient level, were used to reduce sensitivity to potential misspecification of that assumption.

Generalized linear mixed model (GLMM)

As a confirmatory analysis, GLMM with a binomial family, a logit link, and patient-specific random intercepts was fitted using the same set of independent and dependent variables. Fixed effect covariates included age, sex, number of comorbidities, discharge service, polypharmacy, LOS, and major warfarin pDDIs. Random effects were modeled with each participant as a random effect.

Handling of missing data

There were no missing values among the pre-specified baseline covariates, resulting in a complete-case analysis for all independent variables. Both the GEE and GLMM frameworks natively accommodated the unbalanced longitudinal follow-up by using all available post-discharge INR observations per participant, despite varying follow-up durations or measurement frequencies. No imputation of missing INR outcomes was performed.

Multicollinearity assessment

Multicollinearity among the seven pre-specified covariates was evaluated using Variance Inflation Factors (VIF) and Condition Indexes. All individual VIF values were low

(maximum VIF = 1.15), and the maximum Condition Index was 12.36, below conservative diagnostic thresholds and confirming the structural stability of the regression models.

Supplementary Tables

Supplementary Table S1. Prevalence of major warfarin pDDIs (*N* = 1222)

Interacting drugs	Frequency	%	Cumulative %
Omeprazole	749	40.05	40.05
Aspirin	274	14.65	54.71
Simvastatin	173	9.25	63.96
Clopidogrel	101	5.40	69.36
Enoxaparin	98	5.24	74.60
Amiodarone	70	3.74	78.34
Amoxicillin/clavulanate	64	3.42	81.76
Allopurinol	49	2.62	84.39
Cefdinir	33	1.76	86.15
Cephalexin	27	1.44	87.59
Ciprofloxacin	22	1.18	88.77
Metronidazole	15	0.80	89.57
Rosuvastatin	15	0.80	90.37
Co-trimoxazole	13	0.70	91.07
Dicloxacillin	13	0.70	91.76
Sertraline	13	0.70	92.46
Naproxen	12	0.64	93.10
Amoxicillin	11	0.59	93.69
Levofloxacin	10	0.53	94.22
Etoricoxib	8	0.43	94.65
Valproic acid	8	0.43	95.08
Celecoxib	7	0.37	95.45
Trazodone	7	0.37	95.83
Azithromycin	6	0.32	96.15
Fluoxetine	6	0.32	96.47
Oseltamivir	6	0.32	96.79
Penicillin V	5	0.27	97.06
Clarithromycin	4	0.21	97.27
Gemfibrozil	4	0.21	97.49
Ibuprofen	4	0.21	97.49
Ticagrelor	4	0.21	97.91
Cilostazol	3	0.16	98.07
Leflunomide	3	0.16	98.24
Methotrexate	3	0.16	98.40
Tenofovir/efavirenz/emtricitabine	3	0.16	98.56
Cefixime	2	0.11	98.66
Cyclophosphamide	2	0.11	98.77
Fenofibrate	2	0.11	98.88
Mefenamic acid	2	0.11	98.98

Mirtazapine	2	0.11	99.09
Voriconazole	2	0.11	99.20
Others	15	0.80	100.00
Total	1,870	100.00	

Supplementary Table S2. Distribution of post-discharge INR categories from repeated INR measurements stratified by baseline characteristics (*N* = 3704 total INR measurements)

Characteristics	Subtherapeutic *n* (%)	Therapeutic *n* (%)	Supratherapeutic *n* (%)	Total INR measurements *n*
**Sex**				
Female	946 (47.4)	622 (31.2)	428 (21.4)	1996
Male	882 (51.6)	511 (29.9)	315 (18.5)	1708
**Age (years)**				
<65	902 (50.7)	560 (31.4)	318 (17.9)	1780
≥65	926 (48.1)	573 (29.8)	425 (22.1)	1924
**Discharge service**				
Medicine	985 (45.0)	713 (32.6)	490 (22.4)	2188
Surgery	843 (55.6)	420 (27.7)	253 (16.7)	1516
**Number of comorbidities**				
<5	991 (53.3)	555 (29.9)	312 (16.8)	1858
≥5	837 (45.3)	578 (31.3)	431 (23.3)	1846
**Polypharmacy**				
No	123 (43.5)	107 (37.8)	53 (18.7)	283
Yes	1705 (49.8)	1026 (30.0)	690 (20.2)	3421
**Length of hospital stay (days)**				
<10	916 (48.3)	594 (31.3)	387 (20.4)	1897
≥10	912 (50.5)	539 (29.8)	356 (19.7)	1807
**Major warfarin pDDIs**				
No	264 (41.3)	240 (37.5)	136 (21.2)	640
Yes	1564 (51.0)	893 (29.2)	607 (19.8)	3064

Data are based on repeated post-discharge INR measurements. Because patients could contribute more than one INR measurement during follow-up, the totals represent the number of INR measurements rather than the number of patients. Percentages are row percentages within each subgroup. These descriptive distributions should be interpreted cautiously and not as patient-level comparisons. INR categories were defined using indication-specific therapeutic ranges: INR 2.5–3.5 for mitral valve replacement and 2.0–3.0 for other indications.

Abbreviations: INR, international normalized ratio; pDDIs, potential drug-drug interactions.

Supplementary Table S3. Sensitivity analyses of the association between covariates and INR control using the GEE model within 30, 60, and 90 days post-discharge.

**Covariates**	**Within 30 days**		**Within 60 days**		**Within 90 days**	
	**aORs (95% CIs)**	***P*-values**	**aORs (95% CIs)**	***P*-values**	**aORs (95% CIs)**	***P*-values**
Male	1.01 (0.82-1.24)	0.962	1.03 (0.86-1.22)	0.774	1.01 (0.86-1.18)	0.912
Age ≥65 years	1.11 (0.89-1.38)	0.338	1.07 (0.89-1.28)	0.471	1.08 (0.92-1.28)	0.346
Discharge from surgery	1.08 (0.86-1.35)	0.495	1.12 (0.92-1.35)	0.258	1.24 (1.05-1.48)	**0.014**
No. of comorbidities ≥5	0.97 (0.77-1.21)	0.772	0.93 (0.77-1.12)	0.425	0.96 (0.81-1.14)	0.654
With polypharmacy	1.22 (0.78-1.91)	0.379	1.42 (1.01-1.99)	**0.044**	1.33 (0.98-1.82)	0.070
LOS ≥10 days	0.97 (0.78-1.19)	0.745	1.01 (0.84-1.20)	0.928	1.04 (0.88-1.22)	0.676
With major pDDIs	1.25 (0.95-1.65)	0.116	1.32 (1.06-1.66)	**0.014**	1.36 (1.11-1.67)	**0.003**

Bold P-values indicate statistical significance at $P < 0.05$.

Abbreviations: aORs, adjusted odds ratio; GEE, Generalized Estimating Equation; LOS, length of stay; pDDIs, potential drug-drug interactions.

Supplementary Table S4. Sensitivity analyses of the association between covariates and INR control using the GLMM model within 30, 60, and 90 days post-discharge.

**Covariates**	**Within 30 days**		**Within 60 days**		**Within 90 days**	
	**aORs (95% CIs)**	**P-values**	**aORs (95% CIs)**	**P-values**	**aORs (95% CIs)**	**P-values**
Male	1.01 (0.78-1.30)	0.960	1.02 (0.84-1.24)	0.810	0.99 (0.83-1.19)	0.957
Age ≥65 years	1.30 (0.98-1.73)	0.074	1.21 (0.98-1.50)	0.073	1.19 (0.98-1.45)	0.075
Discharge from surgery	1.11 (0.84-1.46)	0.456	1.14 (0.92-1.40)	0.234	1.28 (1.05-1.55)	**0.012**
No. of comorbidities ≥5	0.93 (0.70-1.22)	0.602	0.89 (0.72-1.10)	0.282	0.93 (0.77-1.12)	0.441
With polypharmacy	1.23 (0.71-2.10)	0.461	1.38 (0.94-2.03)	0.102	1.31 (0.92-1.87)	0.130
LOS ≥10 days	1.02 (0.78-1.32)	0.907	1.03 (0.87-1.30)	0.551	1.09 (0.91-1.31)	0.357
With major pDDIs	1.25 (0.89-1.76)	0.197	1.32 (1.03-1.70)	**0.031**	1.37 (1.09-1.73)	**0.008**

Bold *P*-values indicate statistical significance at $P < 0.05$.

Abbreviations: aORs, adjusted odds ratio; GLMM, Generalized Linear Mixed Model; LOS, length of stay; pDDIs, potential drug-drug interactions.

Supplementary Table S5. Sensitivity analyses of the association between covariates and INR control using the GEE model based on the number of INR measurements (first 3, first 7, and all INR measurements).

**Covariates**	**First 3 INRs**		**First 7 INRs**		**All INRs**	
	**aORs (95% CIs)**	***P*-values**	**aORs (95% CIs)**	***P*-values**	**aORs (95% CIs)**	***P*-values**
Male	0.96 (0.80-1.15)	0.648	1.01 (0.86-1.18)	0.929	1.01 (0.86-1.19)	0.912
Age ≥65 years	1.05 (0.88-1.26)	0.567	1.09 (0.92-1.29)	0.311	1.08 (0.92-1.28)	0.346
Discharge from surgery	1.18 (0.97-1.42)	0.097	1.24 (1.02-1.48)	**0.014**	1.24 (1.05-1.48)	**0.014**
No. of comorbidities ≥5	0.91 (0.75-1.10)	0.328	0.95 (0.80-1.12)	0.537	0.96 (0.81-1.14)	0.654
With polypharmacy	1.31 (0.92-1.87)	0.135	1.34 (0.98-1.82)	0.066	1.33 (0.98-1.82)	0.070
LOS ≥10 days	1.13 (0.94-1.35)	0.192	1.03 (0.88-1.22)	0.685	1.04 (0.88-1.22)	0.676
With major pDDIs	1.32 (1.05-1.65)	**0.017**	1.35 (1.10-1.66)	**0.004**	1.36 (1.11-1.67)	**0.003**

Bold *P*-values indicate statistical significance at $P < 0.05$.

Abbreviations: aORs, adjusted odds ratio; GEE, Generalized Estimating Equation; LOS, length of stay; pDDIs, potential drug-drug interactions.

Supplementary Table S6. Sensitivity analyses of the association between covariates and INR control using the GLMM model based on the number of INR measurements (first 3, first 7, and all INR measurements).

Covariates	First 3 INRs		First 7 INRs		All INRs	
	**aORs (95% CIs)**	***P*-values**	**aORs (95% CIs)**	***P*-values**	**aORs (95% CIs)**	***P*-values**
Male	0.96 (0.78-1.17)	0.654	1.00 (0.83-1.19)	0.986	0.99 (0.83-1.19)	0.957
Age ≥65 years	1.15 (0.93-1.43)	0.195	1.19 (0.98-1.45)	0.075	1.19 (0.98-1.45)	0.075
Discharge from surgery	1.20 (0.97-1.49)	0.096	1.27 (1.05-1.55)	**0.014**	1.28 (1.05-1.55)	**0.012**
No. of comorbidities ≥5	0.89 (0.72-1.11)	0.298	0.92 (0.76-1.11)	0.384	0.93 (0.77-1.12)	0.441
With polypharmacy	1.31 (0.87-1.97)	0.193	1.32 (0.93-1.88)	0.123	1.31 (0.92-1.87)	0.130
LOS ≥10 days	1.16 (0.95-1.42)	0.150	1.08 (0.90-1.30)	0.386	1.09 (0.91-1.31)	0.357
With major pDDIs	1.36 (1.05-1.75)	**0.019**	1.37 (1.09-1.72)	**0.008**	1.37 (1.09-1.73)	**0.008**

Bold *P*-values indicate statistical significance at $P < 0.05$.

Abbreviations: aORs, adjusted odds ratio; GLMM, Generalized Linear Mixed Model; LOS, length of stay; pDDIs, potential drug-drug interactions.

Supplementary Table S7. Sensitivity analyses of the association between covariates and INR control using the GEE model under expanded therapeutic INR definitions.

**Covariates**	**2.5-3.5 for MVR & 2.0-3.0 otherwise**		**2.3-3.7 for MVR & 1.8-3.2 otherwise**		**2.0-4.0 for MVR & 1.5-3.5 otherwise**	
	**aORs (95% CIs)**	***P*-values**	**aORs (95% CIs)**	***P*-values**	**aORs (95% CIs)**	***P*-values**
Male	1.01 (0.86-1.18)	0.912	1.03 (0.88-1.21)	0.706	0.90 (0.76-1.06)	0.209
Age ≥65 years	1.08 (0.92-1.28)	0.346	1.07 (0.91-1.25)	0.440	1.09 (0.92-1.29)	0.312
Discharge from surgery	1.24 (1.05-1.48)	**0.014**	1.21 (1.02-1.44)	**0.025**	1.14 (0.96-1.37)	0.137
No. of comorbidities ≥5	0.96 (0.81-1.14)	0.654	1.03 (0.88-1.22)	0.692	1.05 (0.88-1.25)	0.576
With polypharmacy	1.33 (0.98-1.82)	0.070	1.21 (0.88-1.65)	0.239	1.20 (0.86-1.68)	0.279
LOS ≥10 days	1.04 (0.88-1.22)	0.676	1.06 (0.90-1.24)	0.482	1.14 (0.97-1.35)	0.111
With major pDDIs	1.36 (1.11-1.67)	**0.003**	1.25 (1.02-1.52)	**0.028**	1.21 (0.99-1.49)	0.067

Bold P values indicate statistical significance at $P < 0.05$.
Abbreviations: aORs, adjusted odds ratio; GEE, Generalized Estimating Equation; LOS, length of stay; pDDIs, potential drug-drug interactions.

Supplementary Table S8. Sensitivity analyses of the association between covariates and INR control using the GLMM under expanded therapeutic INR definitions.

**Covariates**	**INR 2.5-3.5 for MVR & 2.0-3.0 otherwise**		**INR 2.3-3.7 for MVR & 1.8-3.2 otherwise**		**INR 2.0-4.0 for MVR & 1.5-3.5 otherwise**	
	**aORs (95% CIs)**	***P*-values**	**aORs (95% CIs)**	***P*-values**	**aORs (95% CIs)**	***P*-values**
Male	0.99 (0.83-1.19)	0.957	1.02 (0.85-1.22)	0.854	0.86 (0.70-1.04)	0.126
Age ≥65 years	1.19 (0.98-1.45)	0.075	1.12 (0.92-1.37)	0.241	1.12 (0.90-1.39)	0.305
Discharge from surgery	1.28 (1.05-1.55)	**0.012**	1.26 (1.04-1.52)	**0.021**	1.20 (0.97-1.49)	0.087
No. of comorbidities ≥5	0.93 (0.77-1.12)	0.441	1.02 (0.84-1.23)	0.861	1.06 (0.86-1.31)	0.575
With polypharmacy	1.31 (0.92-1.87)	0.130	1.21 (0.85-1.72)	0.296	1.27 (0.85-1.89)	0.242
LOS ≥10 days	1.09 (0.91-1.31)	0.357	1.12 (0.93-1.34)	0.226	1.22 (1.00-1.49)	**0.045**
With major pDDIs	1.37 (1.09-1.73)	**0.008**	1.28 (1.01-1.60)	**0.038**	1.21 (0.94-1.55)	0.141

Bold P values indicate statistical significance at $P < 0.05$.

Abbreviations: aORs, adjusted odds ratio; GLMM, Generalized Linear Mixed Model; LOS, length of stay; pDDIs, potential drug-drug interactions.

Supplementary Table S9. Summary sensitivity analyses for the association between major warfarin pDDIs at discharge and nontherapeutic INR.

Analysis	Adjusted ORs* (95% CIs)	*P*-value
**Standard therapeutic INR definition: 2.0-3.0, or 2.5-3.5 for MVR**		
INR measurements within 30 days post-discharge		
GEE	1.25 (0.95-1.65)	0.116
GLMM	1.25 (0.89-1.76)	0.197
INR measurements within 60 days post-discharge		
GEE	1.32 (1.06-1.66)	0.014
GLMM	1.32 (1.03-1.70)	0.031
INR measurements within 90 days post-discharge		
GEE	1.36 (1.11-1.67)	0.003
GLMM	1.37 (1.09-1.73)	0.008
First 3 consecutive INR measurements		
GEE	1.32 (1.05-1.65)	0.017
GLMM	1.36 (1.05-1.75)	0.019
First 7 consecutive INR measurements		
GEE	1.35 (1.10-1.66)	0.004
GLMM	1.37 (1.09-1.72)	0.008
**Expanded therapeutic INR definition: 1.8-3.2, or 2.3-3.7 for MVR, and INR measurements within 90 days post-discharge**		
GEE	1.25 (1.02-1.52)	0.028
GLMM	1.28 (1.01-1.60)	0.038
**Expanded therapeutic INR definition: 1.5-3.5, or 2.0-4.0 for MVR, and INR measurements within 90 days post-discharge**		
GEE	1.21 (0.99-1.49)	0.067
GLMM	1.21 (0.94-1.55)	0.141

* Adjusted for sex, age, discharge service, no. of comorbidities, the presence of polypharmacy, and length of hospital stay.

Abbreviations: CI, confidence interval; GEE, Generalized Estimating Equation; GLMM, Generalized Linear Mixed Model; INR, international normalized ratio; MVR, mitral valve replacement; OR, odds ratio

Supplementary Figure

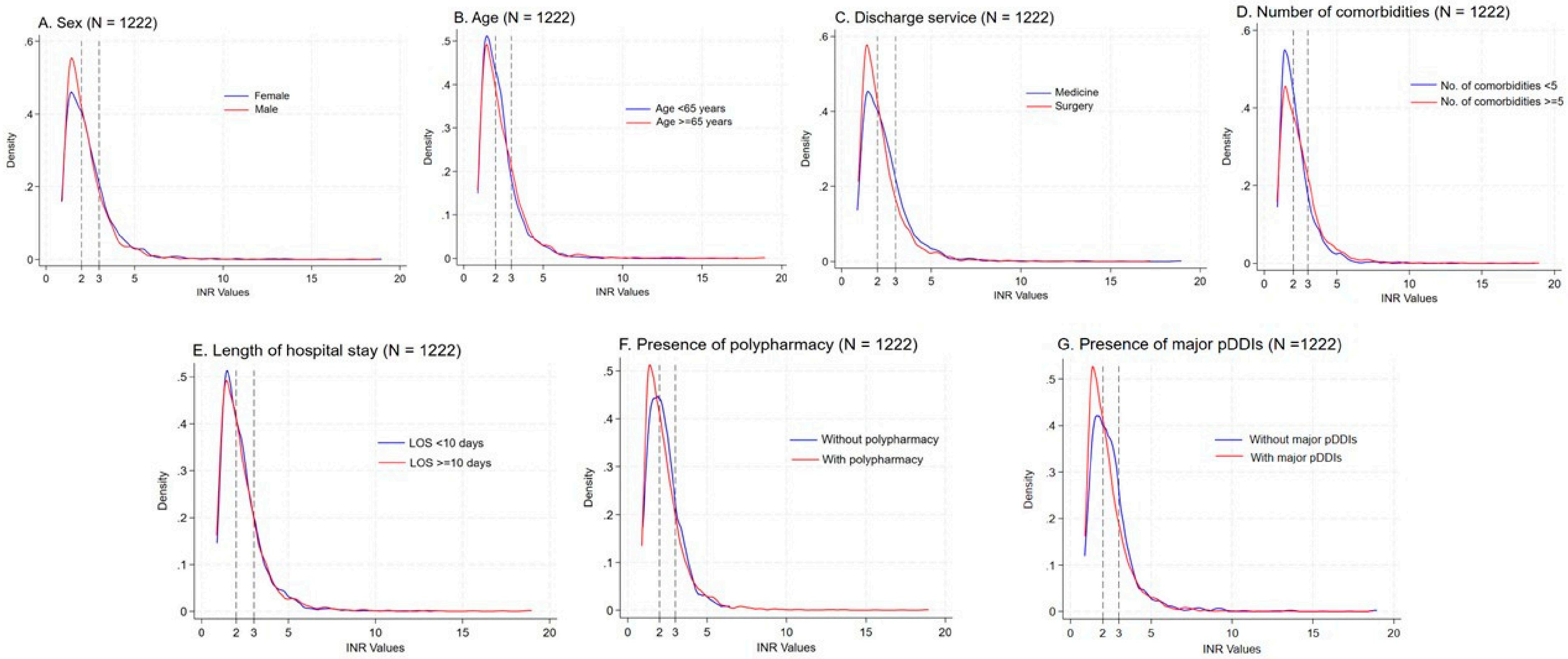

Supplementary Figure S1. Probability density plots of INR values stratified by baseline characteristics. Panel A shows sex (female vs male); Panel B, age group; Panel C, discharge service; Panel D, number of comorbidities; Panel E, length of hospital stay; Panel F, presence of polypharmacy; and Panel G, presence of major warfarin pDDIs. Vertical dashed lines indicate the INR values of 2.0-3.0.
